# Breastfeeding and Environmental Consciousness: A Narrative Review of Environmental Implications and Potential Contributions to Reduce Waste and Energy Consumption

**DOI:** 10.7759/cureus.45878

**Published:** 2023-09-24

**Authors:** Ipsita Mohapatra, Subha R Samantaray

**Affiliations:** 1 Obstetrics and Gynecology, All India Institute of Medical Sciences, Kalyani, Kalyani, IND

**Keywords:** carbon footprint, waste reduction, energy consumption, environmental sustainability, breastfeeding

## Abstract

Breastfeeding is a natural and essential process that not only confers numerous health benefits to infants and mothers but also plays an important role in environmental sustainability. This narrative review explores the environmental implications of breastfeeding in comparison to formula feeding and examines the potential contributions of breastfeeding to reduce waste, energy consumption, and carbon footprint. By exploring the existing literature and research findings, this review sheds light on how breastfeeding aligns with environmental conservation efforts and reinforces the importance of promoting breastfeeding practices for a more sustainable and environment-friendly future.

## Introduction and background

Breastfeeding and formula feeding are two distinct methods of providing nutrition to infants during their early months of life. Both approaches have their own benefits and considerations, impacting the health of the infant, the mother, and the environment.

Breastfeeding is the natural process of feeding an infant with breast milk produced by the mother's mammary glands. Breast milk is a complete and balanced source of nutrition, containing a mix of proteins, fats, carbohydrates, vitamins, minerals, and antibodies in the right proportion that can support the baby's growth and immune system development [1]. It is easily digestible and promotes healthy gut flora, reducing the risk of allergies, infections, and chronic diseases. Breastfeeding is also associated with the development of bonding between mother and infant, and it may contribute to the emotional well-being of both the mother and infant [2]. It is considered environmentally sustainable, as it requires almost nil resources, no packaging, and produces no waste.

Formula feeding involves providing infants with commercially prepared infant formula, which is a substitute for breast milk. Infant formula is carefully manufactured to mimic the nutritional composition of breast milk, but it may not contain all the bioactive components found in breast milk [3]. Formula feeding can provide a suitable alternative when breastfeeding is not possible due to medical reasons or due to personal choices. It can be given by caretakers other than the mother and thus allows flexibility for parents and caretakers.

However, formula feeding may not produce the same immunological benefits as breast milk and can be associated with a slightly increased risk of infections and certain health conditions [4]. Formula feeding has environmental concerns also, including the production, packaging, transportation, and waste generation associated with manufacturing and using infant formula products.

Overall, breastfeeding is often recommended by healthcare professionals as the optimal method of infant nutrition due to its numerous health benefits for both the infant and mother. However, formula feeding can be a necessary and valid choice for some families. The decision between breastfeeding and formula feeding is a personal one, influenced by a range of factors, including medical conditions, lifestyle, cultural norms, occupational compulsions, and personal preferences.

## Review

The importance of environmental consciousness in the context of breastfeeding is intertwined with the urgent need to address global challenges associated with health, climate change, resource depletion, and waste management. Recognizing the environmental impact of infant feeding practices, particularly breastfeeding, is crucial for achieving a more sustainable and resilient future.

Breastfeeding contributes minimally to carbon emissions compared to the energy-intensive processes associated with formula production, including manufacturing, packaging, and transportation [5]. By promoting breastfeeding, we reduce the carbon footprint of infant nutrition, which aligns with efforts to mitigate climate change. Breastfeeding requires no additional resources, such as water for formula preparation or energy for manufacturing. As global resources such as water become scarcer, promoting breastfeeding conserves vital resources and supports sustainable consumption patterns. Formula feeding generates significant waste, including packaging materials, formula cans, and single-use bottles [6]. Breastfeeding eliminates the need for such waste, reducing the burden on landfills and promoting a circular economy. Sustainable infant feeding practices, such as breastfeeding, contribute to a healthier environment. Breast milk is a natural and biologically appropriate source of nutrition, minimizing the pollution and waste associated with formula production and disposal.

Breastfeeding provides infants with optimal nutrition and immune protection, which can be particularly important in resource-limited or disaster-prone areas. Promoting breastfeeding strengthens infant resilience in the face of environmental challenges such as floods and cyclones. Breastfeeding is accessible to all socioeconomic groups, reducing disparities in infant nutrition and health outcomes. By prioritizing breastfeeding, we ensure that access to optimal infant nutrition is not compromised by environmental or economic factors.

Waste generation by formula feeds

The analysis of waste generated by formula feeding reveals a significant environmental impact resulting from the production, packaging, and consumption of infant formula [7]. This waste encompasses various aspects, including packaging materials, single-use bottles, and the energy-intensive manufacturing processes involved. Understanding these factors is crucial for assessing the ecological implications of formula feeding and highlighting the potential benefits of promoting more sustainable infant feeding practices such as breastfeeding.

Infant formula typically comes in cans or plastic containers, often encased in additional packaging materials for branding and information purposes. The production and disposal of these packaging materials contribute to plastic waste, which is a major environmental concern due to its slow degradation and potential harm to ecosystems. Apart from this, formula feeding often involves the use of single-use plastic bottles, nipples, and caps for feeding the baby. These items are discarded after each use, leading to significant plastic waste accumulation. Single-use plastic bottles, nipples, and caps used in formula feeding contribute to plastic pollution in oceans and landfills, negatively impacting marine life and ecosystems [8].

The production, transportation, and disposal of these items contribute to resource consumption and pollution. At the same time, the manufacturing of infant formula involves energy-intensive processes, including milk extraction, drying, blending, and packaging [9]. The energy and resources required for these processes contribute to greenhouse gas emissions and resource depletion, further exacerbating environmental challenges. The energy-intensive processes involved in formula production, including milk extraction, processing, and packaging, contribute to carbon emissions and climate change. The distribution of formula products through global supply chains involves energy-intensive transportation methods, leading to carbon emissions from fossil fuel combustion. The overall carbon footprint of formula feeding is significantly higher than breastfeeding due to the combination of energy-intensive processes and resource consumption.

The transportation and distribution of formula products involve energy consumption and environmental pollution, contributing to air pollution and climate change. Long supply chains and global distribution networks further amplify the environmental footprint associated with formula feeding.

The disposal of formula packaging, bottles, and other related waste contributes to landfill accumulation, further straining waste management systems. Plastics and other non-biodegradable materials can persist in landfills for decades, releasing harmful substances and contributing to environmental degradation [10]. Formula feeding generates substantial packaging waste, including cans, plastic containers, and labels. The production and disposal of these materials contribute to pollution and strain waste management systems [1]. The energy-intensive processes involved in formula production, such as milk extraction and drying contribute to air and water pollution through greenhouse gas emissions, chemical releases, and waste discharge [8].

In addition to environmental concerns, the use of plastic bottles and containers may pose health risks due to the potential leaching of harmful chemicals into the formula [11]. Understanding the waste generated by formula feeding highlights the need for more sustainable infant feeding practices. Promoting breastfeeding, which generates minimal waste and has a lower carbon footprint, can play a significant role in reducing the environmental impact associated with infant nutrition. Additionally, efforts to develop eco-friendly packaging and more sustainable formula production processes can help mitigate the waste generated by formula feeding and contribute to a more environmentally conscious approach to infant nutrition.

The parents who are using formula feeds are encouraged to throw away or discard any leftover prepared formula feed. However, in the case of breastfeeding, there is no such wastage [12].

Breastfeeding stands out as a waste-free feeding method with numerous environmental benefits. Unlike formula feeding, which involves the production, packaging, and disposal of various materials, breastfeeding minimizes waste at every stage. This waste reduction contributes to environmental sustainability and aligns with efforts to address global challenges related to climate change, resource depletion, and pollution. Breastfeeding does not require the use of single-use bottles, nipples, caps, or other disposable feeding equipment. This avoids the accumulation of plastic waste generated by these items.

Energy requirements

Breastfeeding embodies a natural cycle where the mother's body produces milk in response to the baby's needs. This aligns with the principles of a circular economy by minimizing waste and maximizing resource efficiency. Breast milk is uniquely tailored to meet the baby's nutritional needs and supports optimal growth and development. The health benefits of breastfeeding further emphasize its positive impact on the overall well-being of infants.

Breast milk is produced by the mother's body and delivered directly to the baby, without the need for energy-intensive manufacturing processes. This reduces the energy consumption and associated carbon emissions that result from formula production. Breastfeeding relies on the mother's body to provide nutrition, eliminating the need for resource-intensive processes such as water extraction, formula production, and transportation. This sustainable resource use helps conserve vital natural resources. Breastfeeding eliminates the need for packaging materials, such as cans, plastic containers, and boxes, which are common with formula feeding. This reduction in packaging waste significantly reduces the environmental burden associated with infant nutrition. The absence of manufacturing and transportation processes in breastfeeding results in a lower carbon footprint compared to formula feeding. This contributes to efforts to mitigate climate change and reduce greenhouse gas emissions.

The energy requirements for producing formula milk compared to breastfeeding highlight the stark contrast between the two feeding methods. Formula manufacturing involves energy-intensive processes at multiple stages, from raw material extraction to distribution, contributing to higher energy consumption and greenhouse gas emissions [6]. In contrast; breastfeeding relies on the mother's body to produce milk, resulting in significantly lower energy expenditure.

Formula milk requires the cultivation of crops (such as dairy cow feed) or the rearing of livestock (for milk production), both of which involve energy-intensive activities such as planting, harvesting, and animal care [6]. These processes demand machinery, fertilizers, pesticides, and energy for transportation. After milk is collected from cows, it undergoes pasteurization, homogenization, and drying to create the powdered or liquid formula. The processing steps involve heating, cooling, and mechanical operations that consume substantial amounts of energy. Formulas are produced in specialized facilities that require energy for equipment operation, mixing, and packaging. Packaging materials, such as cans and labels, are produced, contributing further to energy consumption. Formula products are packaged and transported to various locations, often involving long supply chains and global distribution networks. Thus, energy is expended in the transportation of raw materials, packaging materials, and finished products.

Pasteurization and sterilization steps are also energy-intensive processes that ensure the safety and shelf life of formula products. These steps involve heating the product to specific temperatures to kill harmful microorganisms. Spray drying is a common method used to transform liquid formula into powdered form [13]. It requires high heat to evaporate the moisture content, resulting in energy consumption. The production of cans, plastic containers, and labels for formula packaging requires energy-intensive manufacturing processes. The distribution of formula products involves energy-intensive transportation, including shipping, trucking, and air travel, contributing to greenhouse gas emissions.

The production of infant formula requires the extraction and processing of natural resources, such as water, energy, and raw materials. These resource-intensive processes contribute to resource depletion and the strain on ecosystems. Formula production requires significant resource consumption, including water for irrigation and animal hydration, exacerbating water scarcity and resource depletion [14].

Equitable access and sustainability

Breastfeeding's sustainability lies in its inherent ability to minimize resource consumption at every stage of the feeding process. Breastfeeding emerges as a sustainable and natural feeding option that aligns with principles of ecological stewardship and resource conservation. Breastfeeding reduces the demand for land resources required for livestock grazing and crop cultivation for formula production. This indirectly helps preserve ecosystems and prevents deforestation. This helps in sustainable land use. As it is an energy-efficient method, it is more sustainable.

Breastfeeding is accessible to mothers across diverse socioeconomic backgrounds, promoting equitable access to optimal infant nutrition. By endorsing breastfeeding, we support sustainability without compromising health. Breastfeeding generates no packaging waste, as breast milk is directly delivered from the mother to the baby. This reduction in waste aligns with sustainability goals by reducing the burden on waste management systems.

Formula production requires substantial water for irrigation, livestock hydration, and manufacturing processes. Breastfeeding eliminates the need for water-intensive activities associated with formula production. Moreover, breast milk requires no packaging or transportation, reducing the carbon emissions associated with the production and distribution of formula packaging materials. All these factors make the practice of breastfeeding more sustainable as compared to formula feeding.

Land use and ecosystem impact

The rearing of livestock for milk production contributes to deforestation, habitat destruction, and soil degradation, impacting biodiversity and ecosystem health [15]. The cultivation of crops for animal feed (for dairy cows) or formula ingredients further intensifies land use, leading to increased agricultural pressure on natural landscapes [15]. Demand for land for livestock grazing and crop cultivation can drive deforestation, contributing to a loss of carbon sinks and biodiversity. Land conversion for formula-related agricultural activities can lead to habitat fragmentation, disrupting ecosystems and endangering species [16].

Exploration of the influence of cultural norms and marketing practices

Infant feeding choices are profoundly shaped by cultural norms, marketing strategies, and policies that influence parental decisions. Understanding these influences is essential for comprehending why some families opt for formula feeding over breastfeeding.

Cultural norms play a significant role in shaping perceptions of infant feeding. In some societies, breastfeeding might be deeply ingrained as a traditional practice, while, in others, formula feeding might be considered more modern or convenient [17]. Societal attitudes toward breastfeeding in public or workplace support can also influence maternal choices.

At the same time, marketing practices by formula manufacturers can sway parental preferences [18]. Aggressive advertising, endorsements by healthcare professionals, and promotional materials can create an impression of formula feeding as equivalent or even superior to breastfeeding. Such strategies can create a demand for formula products, influencing parents' decisions.

Barriers to effective breastfeeding implementation

Lack of supportive environments: Limited workplace support for breastfeeding, inadequate public spaces for nursing, and lack of breastfeeding-friendly policies can discourage mothers from breastfeeding, especially when they return to work.

Socioeconomic factors: Financial constraints, lack of access to healthcare, and affordability of formula can influence feeding choices. Breastfeeding may be perceived as a luxury that only some can afford.

Medicalization and lack of education: Medical interventions, formula recommendations by healthcare professionals, and lack of comprehensive breastfeeding education can undermine mothers' confidence in their ability to breastfeed.

Inadequate parental leave: Short or unpaid parental leave policies can hinder the establishment of breastfeeding routines and make it difficult for mothers to continue breastfeeding after returning to work.

Policies and initiatives promoting breastfeeding as an environmentally friendly option: Promoting breastfeeding as an environmentally friendly option requires a combination of policy efforts, educational campaigns, and community support. Many countries have implemented national breastfeeding promotion campaigns that emphasize the health benefits of breastfeeding for both mother and child. These campaigns can also raise awareness about the environmental advantages of breastfeeding as a waste-free and resource-efficient choice. The World Breastfeeding Week celebrated in the first week of August every year is a step taken by the government to promote breastfeeding all over the world. This year’s slogan for the breastfeeding week observation was “Let’s make breastfeeding and work, work!" to promote breastfeeding among working women.

Policies mandating workplace lactation accommodations can facilitate breastfeeding continuation for working mothers. By enabling mothers to breastfeed or express milk at the workplace, these policies promote breastfeeding's environmental benefits by reducing the need for formula and associated waste [19].

Hospitals and healthcare facilities that adopt the Baby-Friendly Hospital Initiative guidelines encourage breastfeeding initiation and support. These initiatives promote breastfeeding as a natural and sustainable feeding method, aligning with the goal of reducing formula consumption [20].

Policies that provide longer maternity leave periods support breastfeeding continuation and contribute to reducing formula usage. Extended maternity leave can help mothers establish breastfeeding routines and promote infant health [21]. Community-based educational programs that emphasize the environmental benefits of breastfeeding alongside its health advantages can empower families to make informed and sustainable feeding choices [22]. Some countries have implemented taxation or strict regulation of formula marketing to curb aggressive advertising practices and promote breastfeeding as the optimal choice.

Strategies to overcome barriers and promote eco-friendly infant feeding practices

Comprehensive education and awareness campaigns, which are evidence-based, culturally sensitive educational campaigns highlighting the environmental benefits of breastfeeding, should be implemented. Healthcare professionals, communities, and media should be engaged to dispel myths and promote informed decision-making to mothers and families.

Stricter regulations on formula advertising and marketing practices should be enforced, ensuring that information is accurate and unbiased and does not undermine breastfeeding. Advocating workplace policies that provide lactation accommodations, flexible work hours, and parental leave should be initiated. Working mothers should be empowered and encouraged to continue breastfeeding and express milk while maintaining their careers [19].

Community can establish breastfeeding support groups and networks that provide guidance, counseling, and a sense of community for breastfeeding mothers. Peer support can help overcome challenges and normalize breastfeeding [23]. Training the healthcare professionals to provide accurate and supportive breastfeeding guidance. A collaborative approach that involves doctors, nurses, midwives, and lactation consultants should be encouraged. Comprehensive maternity and parental leave policies that allow mothers adequate time to establish breastfeeding and support families in making eco-friendly feeding choices can be encouraged.

## Conclusions

Education about the environmental benefits of breastfeeding can raise awareness and educate individuals about the broader impact of their choices. This awareness can extend to other aspects of sustainable living. By supporting breastfeeding and making people aware of the benefits of breastfeeding, we contribute to the long-term health and well-being of both infants and mothers. A healthy population is better equipped to address and adapt to global challenges.
